# A graph convolutional network with dynamic weight fusion of multi-scale local features for diabetic retinopathy grading

**DOI:** 10.1038/s41598-024-56389-4

**Published:** 2024-03-09

**Authors:** Yipeng Wang, Liejun Wang, Zhiqing Guo, Shiji Song, Yanhong Li

**Affiliations:** 1 School of Computer Science and Technology, Xinjiang University, Urumqi, 830046 China; 2 Department of Automation, Tsinghua University, Beijing, 100084 China

**Keywords:** Computational biology and bioinformatics, Machine learning

## Abstract

Diabetic retinopathy (DR) is a serious ocular complication that can pose a serious risk to a patient’s vision and overall health. Currently, the automatic grading of DR is mainly using deep learning techniques. However, the lesion information in DR images is complex, variable in shape and size, and randomly distributed in the images, which leads to some shortcomings of the current research methods, i.e., it is difficult to effectively extract the information of these various features, and it is difficult to establish the connection between the lesion information in different regions. To address these shortcomings, we design a multi-scale dynamic fusion (MSDF) module and combine it with graph convolution operations to propose a multi-scale dynamic graph convolutional network (MDGNet) in this paper. MDGNet firstly uses convolution kernels with different sizes to extract features with different shapes and sizes in the lesion regions, and then automatically learns the corresponding weights for feature fusion according to the contribution of different features to model grading. Finally, the graph convolution operation is used to link the lesion features in different regions. As a result, our proposed method can effectively combine local and global features, which is beneficial for the correct DR grading. We evaluate the effectiveness of method on two publicly available datasets, namely APTOS and DDR. Extensive experiments demonstrate that our proposed MDGNet achieves the best grading results on APTOS and DDR, and is more accurate and diverse for the extraction of lesion information.

## Introduction

Diabetic retinopathy (DR) is a prominent long-term complication caused by diabetes mellitus. DR is one of the four major blinding eye diseases by damage the retinal blood vessels leading to visual impairment and blindness. Based on the presence or absence of microaneurysms, blood spots, exudates, and other lesion features, DR is mainly classified into five different levels of degree, i.e., no DR, mild DR, moderate DR, severe DR, and proliferative DR (PDR)^1,2^. In Table 1 we list the characteristics of the lesions in each stage of DR. Statistically, about 30% of diabetic patients will eventually develop DR and it is usually not detected until the retinal abnormality reaches a stage where treatment is ineffective or impractical. Therefore, regular screening of diabetic patients is essential. However, in clinical practice, manual diagnosis of DR is time-consuming and prone to human error, especially in the early stages when the lesions associated with DR are often small and difficult to recognize. In addition, as people’s standard of living improves, the intake of high-sugar and high-fat foods has increased dramatically without their knowledge, leading to a surge in the number of diabetic patients, while the shortage of specialized ophthalmologists will result in insufficient professionals to examine all at-risk individuals. For this reason, automated detection and diagnosis of DR are in line with the current times. The automatic grading of DR based on deep learning can not only reduce the workload of medical personnel, but also give more objective and realistic diagnostic results, which has a great positive effect on the diagnosis and treatment of DR.

**Table 1:** The basis for judging five DR categories.

DR Grade	Lesion characterisation
0: No DR	No lesions
1: Mild DR	Presence of microangiomas
2: Moderate DR	In addition to microhaemangiomas, small haemorrhagic spots and exudates are present
3: Severe DR	Patients with>20 hemorrhages in at least one quadrant, vein bead-like changes in at least two quadrants, or intraretinal microvascular abnormalities in at least one quadrant
4: PDR	Presence of neovascularisation, vitreous haemorrhage and proliferative lesions

**Figure 1:**
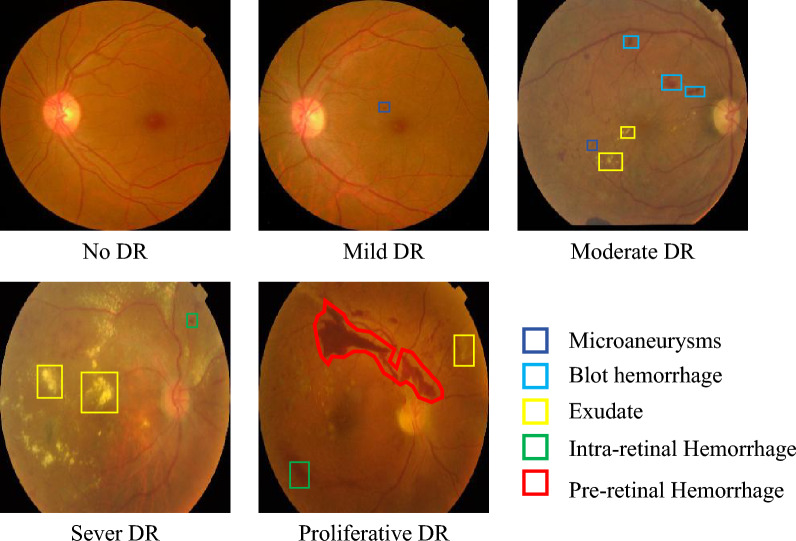
The five severity levels of DR. We marked the different lesion information in the picture with different colored boxes (images from APTOS dataset).

Current methods for DR detection and grading are based on Convolutional Neural Networks (CNNs) and Transformer methods. However, some of the classical network structures are usually designed for natural image recognition, and it is difficult to obtain satisfactory results when applied directly to medical image analysis such as DR detection. Compared to natural images, medical images are very different. Taking fundus images used for DR detection as an example, as shown in Fig. 1, we can find that the lesion features used to diagnose the DR category are not only of diverse shapes but also the vast majority of them are inconspicuous and very similar to the background information. Consequently, it is difficult to directly apply traditional convolutional neural networks to DR grading to extract lesion information and integrate feature information from different locations. The main reason why DR can be accurately detected and graded is that lesion features such as microaneurysms, blood spots, exudates, etc., can be extracted and identified. Current methods usually perform a pre-processing of the image before inputting it into the network model to extract richer lesion features. For example, Ashwini and Dash^3^ used Contrast Limited Adaptive Histogram Equalization (CLAHE) and sampling techniques to process the training data. Liu et al.^4^ proposed a data denoising method for ultra-wide-field (UWF) images and used a data enhancement method to increase its contrast and brightness to improve the grading effect. However, these methods do not improve the feature extraction capability of the model.

There are also some improvements in the model for DR grading. Hou et al.^5^ proposed a Cross-Field Transformer (CrossFiT), which can effectively use dual-field correspondence to improve the DR grading performance. However, these methods have some problems because the features of DR are complex and variable, and it is difficult for a single-scale feature extractor to accurately extract all the lesion features. Moreover, these features are randomly distributed over the whole fundus image, and local and global features must be combined to achieve better DR grading performance. Based on the above description, we propose a multi-scale dynamic fusion (MSDF) module, which is not only capable of extracting lesion features of different sizes but also learns the corresponding weights for feature fusion based on the importance of different features. To interact with local and global features, we combine this module with graph convolution to design a multi-scale dynamic graph convolutional network (MDGNet). In summary, the contributions made in this paper are as follows:

1. Based on the characteristics of DR image lesion features, we propose a multi-scale dynamic fusion (MSDF) module, which not only extracts feature information of different shapes and sizes, but also learns dynamic weights for feature fusion according to the importance of different features to model grading.
2. We propose an MDGNet that effectively combines the advantages of convolution and graph convolution. Firstly, the model has a strong local feature extraction capability and can extract information about lesions with different shapes. Second, the model also has the ability to capture long-range dependencies and can fuse lesion information from different regions of the image.
3. We conduct extensive experiments on two public datasets APTOS and DDR to verify the effectiveness of module. And we also conduct comparative experiments with other state-of-the-art models to demonstrate that our method has a good DR grading capability.

## Related work

In recent years, artificial intelligence (AI) has been referenced in various aspects of the medical field. For example, Zhang et al.^6^ proposed an MLP-based model for the classification of COVID-19 and skin diseases. Wang et al.^7^ proposed an arterial and venous de-entanglement network (AVDNet), which is the first work to segment coronary arteries and veins at the same time. Dai et al.^8^ proposed a new medical image Few-shot classification method for solving the medical image number less problem. In this section, we focus on methods for DR detection and grading.

The detection and grading of DR is not a recent concern, there have been many early studies on the subject. Early studies on DR detection were usually traditional machine learning methods. The extraction of features usually needs to be performed manually, where the extracted features are recognized for grading. Akram et al.^9^ proposed a three-phase system for the early detection of microaneurysms (MAs). In the first stage, the system extracts all possible candidate regions for MAs present in the retinal image. In the second stage, feature vectors are formulated for each region based on certain characteristics (i.e. shape, color, intensity, and statistics). In the third stage, these feature vectors are identified using the proposed hybrid Gaussian Mixture Model (GMM) and Support Vector Machine (SVM) classifiers. Akram et al.^10^ used a similar approach for detecting retinopathy. Verma et al.^11^ proposed a Random Forest based approach to classify the different stages of eye disease based on the area and perimeter of the blood vessels and hemorrhages in the retinal image. Kar et al.^12^ proposed a DR detection scheme with four main stages: vessel extraction and disc removal, pre-processing, candidate lesion detection, and post-processing. Welikala et al.^13^ proposed an automated detection of new vessels from retinal images for identifying proliferative diabetic retinopathy. However, the hand-crafted features are highly dependent on the experience of the designer and only partially describe certain lesions with a fixed pattern. Since DR contains complex lesions with diverse appearances and spatial distributions, there is an urgent need to propose more advanced methods with high generalization capabilities and sufficient robustness.

To address the drawbacks of manual feature extraction, researchers have utilized CNNs to automatically extract and fuse task-relevant features, thus circumventing the limitations of traditional hand-crafted feature-based approaches. For example, Gargeya et al.^14^ used Resnet as a feature extractor to extract features of lesion information related to DR and applied a decision tree to determine the presence of DR in a patient. Shanthi et al.^15^ improved the Alexnet network by applying appropriate pooling, softmax, and Relu, and achieved better DR grading accuracy. Gayathri et al.^16^ used a simple 6-layer convolutional layer CNN for DR feature extraction and fed their features to different machine learning classifiers (SVM, AdaBoost, Naive Bayes, Random Forest, and J48) for grading. Hemanth et al.^17^ combined histogram equalization and contrast-limited adaptive histogram equalization image processing techniques with deep learning to propose an alternative hybrid solution approach for DR detection. Ayhan et al.^18^ proposed a data-driven approach to quantify the prediction uncertainty of deep neural networks (DNNs), paving the way for a comprehensive treatment of uncertainty in DNN-based diagnostic systems. Saxena et al.^19^ applied an advanced convolutional neural network model for early detection of DR to accelerate the initial screening of DR to meet the future needs of such a large number of diabetic patients. Cao et al.^20^ used the Resnet as a backbone network and enhanced the effect of feature extraction by modifying the residual blocks in it and utilizing the attention mechanism for DR severity grading. Shaik et al.^21^ devised a method called Hinge Attention Network (HA-Net), which uses a pre-trained VGG16 to extract feature information, and then combines multiple attention mechanisms to achieve a high correct rate of DR grading. Li et al.^22^ proposed a novel cross-disease attention network (CANet), which enables joint classification of DR and DME by exploring the internal connection between diabetic retinopathy (DR) and diabetic macular edema (DME, a complication of DR). Zhao et al.^23^ proposed an in addition to a deep learning architecture called BiRA- Net deep learning architecture, which combines an attention model for feature extraction and a bilinear model for fine-grained classification. In addition, Zhao et al.^23^ proposed a “graded loss” loss function to improve the training convergence of the proposed model. Canayaz et al.^24^ designed a method based on the selection and packing of fundus images. The method first eliminates the useless dark areas in the image by image processing techniques, then wraps the features extracted by EfficientNet^25^ and DenseNet^26^, selects the most effective features, and finally classifies them by vector machine and random forest machine learning methods.

With the development of hardware technology and deep learning, deep learning-based DR grading methods have obvious advantages and have become the mainstream methods for DR grading tasks. However, the current approach still has some limitations. Firstly, it does not distinguish between natural images and fundus images, and the network architecture specialized for natural image classification is directly applied to fundus images. Secondly, it cannot flexibly and effectively extract the information features of lesions of different sizes and shapes. Finally, there is no combination of local and global features. There are several difficulties in performing DR grading. Firstly, the lesion regions among the DR images are of various shapes, to address this problem, we propose a multi-scale dynamic fusion (MSDF) module, which can extract lesion features of different sizes and shapes, and dynamically perform feature fusion according to the importance of their lesion information. Second, the lesion part is randomly distributed in the whole image, and it is difficult to interact with the lesion information at different locations. To solve this problem, we introduce graph convolution into DR grading. In summary, we propose a new model MDGNet in this paper, which combines the advantages of convolution and graph convolution to achieve accurate DR grading.

## Method

### Network architecture

**Figure 2:**
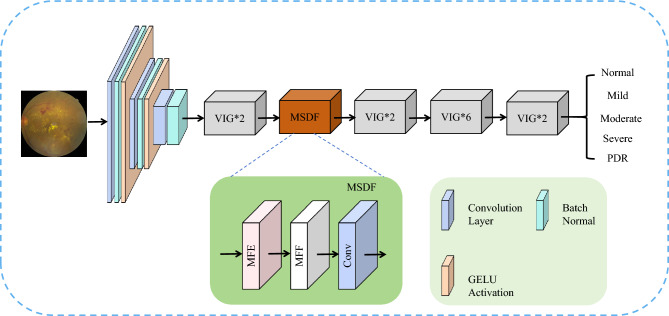
Overall architecture of the MDGNet model.

Features such as microaneurysms, blood spots, exudates, and neovascularisation in DR fundus images are the main information for the correct DR grading. This requires the model to be able to extract not only different types of lesion features at different scales but also to consider the interaction between different local features. To address these issues, we designed a multi-scale dynamic fusion (MSDF) module. It can not only extract multi-scale features but also perform feature fusion based on dynamic learning weights. We combine it with a graph convolution network to propose a novel model MDGNet. The structure of the model is shown in Fig. 2. First, the original input image undergoes multiple convolutions, BatchNorm, and activation to reduce the feature resolution and increase the feature dimensionality. Then it goes through a graph convolution module ViG^27^ for feature convergence. Next is the MSDF module. This module consists of a multi-scale feature extraction (MFE) module, a multi-feature fusion (MFF) module, and a convolutional layer. MSDF module is capable of extracting feature information at different scales and dynamically learning the corresponding weights for feature fusion through the connection between multiple features. This is followed by multiple ViG modules to achieve global interaction of different local features. Finally, the DR grading results of the model are input.

### ViG block

**Figure 3:**
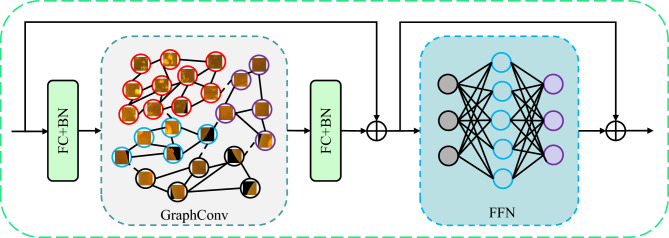
The structure of ViG block.

Visual GNN (ViG)^27^ represented images as graph structures that can extract features at the graph level for visual tasks. As shown in Fig. 3, the ViG block consists of two parts, a graph convolution module for aggregating and updating the graph information, and an feedforward network (FFN) module for transforming the node’s feature information. The main operation flow of the ViG is as follows. For feature $X\in R^{N \times D}$ we first use the k-nearest neighbor algorithm to determine the neighbors of each node and construct it as a graph. Secondly, the graph convolution operation is performed to aggregate the information of each neighboring node. Finally, the node’s characteristic information is transformed to enhance the node’s nonlinear expression ability.

1 $$\begin{aligned}X^\prime &= FC(GraphConv(FC(X))) + X\\ Y &= FC(\sigma (FC(X^\prime ))) + X^\prime  \end{aligned}$$

where X is the input feature, GraphConv is the graph convolution operation, and Y is the output feature, after each FC there is a BN. In this paper, we use the max-relative graph convolution^28^ operation, which is calculated as follows.

2 $$\begin{aligned} y&= GraphConv(x_i)\\&= \sigma (FC(x_i, max(\{x_i-x_j|x_j \in N(x_i)\})))  \end{aligned}$$

where $x_i$ is the node to be graph-convoluted, $N(x_i)$ denotes all neighboring nodes of $x_i$, $max(\cdot )$ serves to take the maximum value.

### MFE block

**Figure 4:**
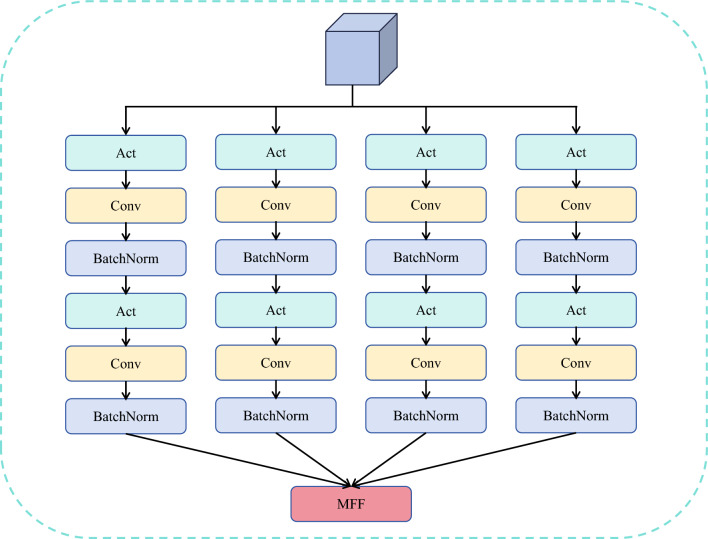
The structure of MFE block.

Extracting multi-scale features is the key to DR image lesion information extraction. As shown in Fig. 1, we can find that among the DR fundus images, the shapes and sizes of microaneurysms, blood spots, exudates, and other feature information are not fixed. Moreover, for a patient with DR severity, his fundus image has multiple types of symptoms and contains more complex feature information. To capture more detailed lesion features at each information scale, we designed a multi-scale feature extraction (MFE) module. Our MFE module uses four branches, which can learn feature information at different scales and classes of lesions in different complex DR images. As shown in Fig. 4, the MFE mainly consists of convolutional kernels of different sizes and features from different sizes of convolutional kernels are fused to obtain the final result. Convolutional kernels of different sizes allow the model to focus on different scale information in the image simultaneously. Small-scale convolutional kernels can be used to capture details and tiny lesions, while large-scale convolutional kernels can be used to capture larger and global lesions. This helps in understanding the lesions in the image in a more comprehensive way. The MFE operates as follows. Firstly the features from the upper layer are subjected to an activation operation to enhance the representation and learning ability of the module. Then different types and scales of information are extracted through convolution kernels of different sizes. We finally use a 1x1 size convolution to perform dimensional transformation and increase the feature representation capability of the module. To make the model more block convergent and the features are in a fixed distribution, we use BN after each convolutional layer. The specific operation of the whole module can be used as the following representation.

3 $$\begin{gathered}   X_{1}  = BN(Conv_{1} (GELU(BN(Conv_{1} (GELU(X)))))) \hfill \\   X_{2}  = BN(Conv_{1} (GELU(BN(Conv_{3} (GELU(X)))))) \hfill \\   X_{3}  = BN(Conv_{1} (GELU(BN(Conv_{5} (GELU(X)))))) \hfill \\   X_{4}  = BN(Conv_{1} (GELU(BN(Conv_{7} (GELU(X)))))) \hfill \\   X_{{output}}  = MFF\left( {X_{1} ,X_{2} ,X_{3} ,X_{4} } \right) \hfill \\  \end{gathered}$$

where X is the input feature, $Conv_i$ denotes the convolution operation with convolution kernel size i, BN denotes regularisation and $X_{output}$ is the output result.

### MFF block

Feature fusion is another important operation of many deep learning-based methods, which facilitates the full integration of different levels of information to improve the representation of features and enhance model performance. For DR grading, the extent to which different lesion information contributes to the final result is also generally different. For example, for the PDR category, although it also has features such as exudates and hemorrhages, the most significant feature is the formation of neovascularization. Therefore, when facing the PDR category, we should pay more attention to these feature information of neovascularization. CBAM^29^ proposed channel attention and spatial attention, which enable the model to focus on the main feature information in the spatial and channel directions. Based on the above-mentioned, we design a multi-feature fusion (MFF) module. This module not only enhances important features on the channel but also adaptively generates appropriate weights for feature fusion based on the importance between different features. Feature fusion with dynamic weights has several advantages. Firstly, the feature fusion process with dynamic weights can reduce information loss because the model can flexibly adjust the contribution of each channel to ensure that critical information is not overlooked. Second, it can also improve the robustness of the model so that it can work under various environmental conditions. Because it can adaptively cope with noise, light variations, and other disturbances, it is more useful in realistic clinical settings. Finally, with adaptive weight learning, the model may be better able to generalize to new and unseen data. This is important for diagnosis and prediction in medical image analysis, as image data can vary greatly.

**Figure 5:**
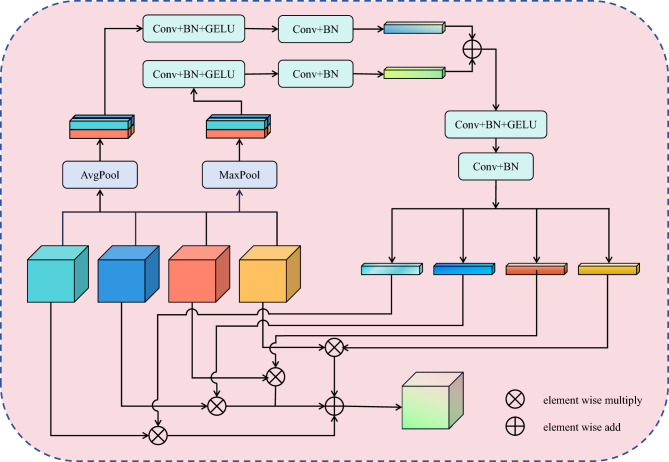
The structure of MFF block.

Fig. 5 shows the specific details of the MFF module, which performs dynamic weight fusion of multi-scale features from the MFE module. First, we perform average pooling and maximum pooling on all feature maps. Average pooling not only captures the global information in the input feature maps but also reduces the noise impact of the features and helps to smooth the features in the image. Maximum pooling highlights the most salient features in the image and allows the module to adaptively determine which features are most important for a particular task. This allows the model to automatically select key features based on the needs of the task. Then the average pooling of multiple features is stitched together and undergoes two convolutional operations to interact information about the different average pooled features. Similarly, the same process is performed for multiple maximum pooled features. However, the pooling operation has some shortcomings. Average pooling may result in some important feature information being averaged or lost. Maximum pooling only focuses on the maximum value of the features, which may lead to some useful details being ignored, thus degrading the performance of the model, especially in some tasks that require global information. Therefore, we then combine the processed average pooled features with the maximum pooled features to generate the final weighting information after an MLP containing a hidden layer. This weighting information has the following advantages. First, the module can adaptively determine the importance of each feature channel. This allows the model to better focus on feature channels that are critical to the task. Second, average pooling smooths all channels, and maximum pooling focuses on important features, and this diversity helps the model to better adapt to different data distributions and tasks. Finally, adaptive learning of the weights of each feature map enables dynamic fusion of information from different modules, which improves the performance and flexibility of the model. The implementation details of the MFF module are as follows.

4 $$ \begin{gathered}   X_{{avg}}  = Conv(GELU(Conv(AvgPool(X_{1} ,X_{2} ,X_{3} ,X_{4} )))) \hfill \\   X_{{max}}  = Conv(GELU(Conv(MaxPool(X_{1} ,X_{2} ,X_{3} ,X_{4} )))) \hfill \\   X_{{weight}}  = MLP\left( {X_{{avg}}  + X_{{max}} } \right) \hfill \\  \end{gathered}  $$

where $X_1,X_2,X_3,X_4 \in R^{(B,C,H,W)}$ are the features to be fused and $X_{weight}$ is the learned weight information. Subsequently, $X_{weight}$ is dimensionally transformed and then fused with the corresponding features.

5 $$ \begin{gathered}   X_{{w1}} ,X_{{w2}} ,X_{{w3}} ,X_{{w4}}  = Softmax\left( {Reshape\left( {X_{{weight}} } \right)} \right) \hfill \\   X_{{output}}  = X_{{w1}} *X_{1}  + X_{{w2}} *X_{2}  + X_{{w3}} *X_{3}  + X_{{w4}} *X_{4}  \hfill \\  \end{gathered}  $$

### Evaluation metrics and loss function

In this paper, we use the following metrics to evaluate the effectiveness of our model.

6 $$\begin{aligned} Acc = \frac{TP+TN}{TP+TN+FN+FP} \end{aligned}$$

7 $$\begin{aligned} Precision = \frac{TP}{TP+FP} \end{aligned}$$

8 $$\begin{aligned} Recall = \frac{TP}{TP+FN} \end{aligned}$$

9 $$\begin{aligned} F1 = \frac{Precision\times Recall\times 2}{Precision+Recall} \end{aligned}$$

where TP is the correctly categorized positive sample, TN is the correctly categorized negative sample, FN is the incorrectly categorized positive sample and FP is the incorrectly categorized negative sample. We also added ROC curves and AUC to evaluate the DR grading performance of our method. Since we use a multi-category dataset in this paper, when we calculate these metrics, we first convert the multi-category to multiple binary problems before calculating them, and then finally average them.

In this paper we use the cross-entropy loss function.

10 $$\begin{aligned} loss = \frac{1}{N}\sum _{i}^N\sum _{c}^My_{ic}\log (p_{ic}) \end{aligned}$$

where $p_{ic}$denotes the predicted probability that sample i belongs to category c; $y_{ic}$ is a sign function. If the true category of sample i is equal to c take 1, otherwise take 0; M is the number of categories; N is the number of samples.

## Experiments

### Datasets

In this paper, we use two publicly available fundus image datasets APTOS and DDR. Specific details of the two datasets are given below.

#### APTOS dataset^30^

**Figure 6:**
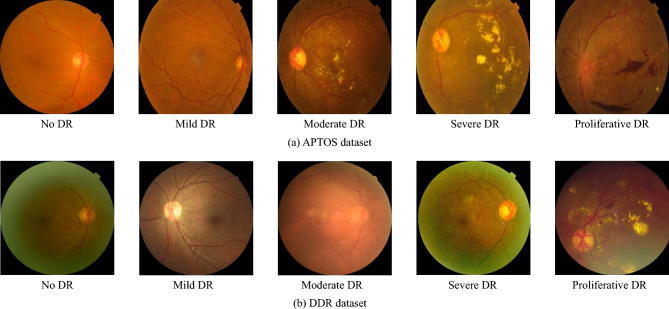
Sample presentation of two datasets. (**a**) and (**b**) show the five categories of the APTOS dataset and the DDR dataset, respectively.

This dataset is provided by the Asia Pacific Tele-Ophthalmology Society for the 2019 Kaggle Blindness Detection Competition. The APTOS dataset consists of 3662 images and these images are categorized into five categories based on the International Clinical Diabetic Retinopathy, which are no DR, mild DR, moderate DR, severe DR, and proliferative DR. In Table 2 and Fig. 6, we give the number of images and image samples for each category respectively.

#### DDR dataset^31^

Dataset for Diabetic Retinopathy (DDR) was collected from 147 hospitals in 23 provinces in China between 2016 and 2018. The DDR contains a total of 13673 fundus images. Since the DDR data collection came from multiple hospitals, the images were taken by 42 different fundus cameras. The images were then classified by seven professionals into six categories according to the International Classification of Diabetic Retinopathy: normal, mild DR, moderate DR, severe DR, proliferative DR, and unclassifiable. The unclassifiable is defined as those images in which the imaging quality is poor and the lesion is not visible. Therefore, in the experiments of this paper, we exclude the unclassifiable category and use only the five categories of normal, mild DR, moderate DR, severe DR, and proliferative DR images. These five categories consist of a total of 12,522 images, and in Fig. 6 we list examples of images from these five categories.

In this paper, we divide the dataset into a training set and test set according to 4:1, and the specific details of the division of the two datasets are shown in Table 2.

**Table 2:** Division of the two datasets.

Datasets		Normal	Mild	Moderate	Severre	PDR	Total
APTOS	Train	1444	296	799	154	236	2929
	Test	361	74	200	39	59	733
	Total	1805	370	999	193	295	3622
DDR	Train	5012	504	3581	188	730	10015
	Test	1254	126	896	48	183	2507
	Total	6266	630	4477	236	913	12522

### Experimental setting

In this paper, the resolution size of 224x224 is used for all our model inputs where not explicitly stated. The optimizer we used is AdamW, Weight decay is set to 0.005, the learning rate is 0.0001, and the data enhancement methods of random clipping, flipping, and ColorJitter are used to alleviate the model overfitting problem during the training process. The batch size of training is 64, and the learning rate decay strategy of cosine annealing is adopted. Finally, all the experiments in this paper were conducted under python 3.6, torch 1.10.0, and NVIDIA TITAN RTX.

### Comparative experiments

In this section, we focus on verifying the validity and superiority of our proposed model MDGNet for DR grading. Firstly, we mainly compare with some state-of-the-art generic models, which are Resnet50^32^, Densenet121^26^, Res2Net^33^, Swin^34^, FasterNet^35^, SMT^36^, FasterViT^37^, CoCs^38^, ViG^27^. For a valid and fair comparison, we use the same experimental setup and data enhancement methods. Subsequently, we also analyze the classification effect of our model for each category of the APTOS and DDR datasets, and discover the regions of interest of the model through some visualization methods to better explain and prove the superiority of our model.

#### Results on APTOS dataset

**Figure 7:**
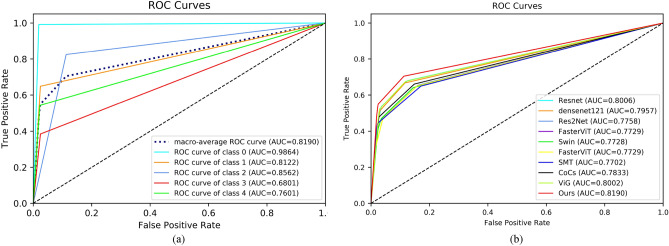
ROC curve on the dataset APTOS. (**a**) is the ROC curve for each category of our model, and (**b**) is the ROC curve for each model.

Table 3 shows the experimental results on the dataset APTOS. In Table 3 we used five evaluation metrics ACC, F1, Precision, Recall, and AUC. From Table 3 we can see that our proposed method achieves the best results, where ACC = 84.31%, F1 = 69.69%, Precision = 72.27%, Recall = 67.84%, and AUC = 81.89%. Compared to the baseline model ViG^27^, our method shows a better improvement in all the metrics, where ACC improves by 1.5%, F1 improves by 3.21%, Precision improves by 2.85%, Recall improves by 3.32%, and AUC improves by 1.88%. Next, we analyze the superiority of our model in terms of multiple metrics. First, above the ACC metrics, our method is generally higher than other methods by more than one percentage point, e.g., 2.87% higher than Swin^34^ and 2.19% higher than Res2Net^33^. This indicates that our method outperforms other models in overall grading. However, since APTOS is a class-imbalanced dataset, we continue our analysis on Recall and AUC metrics. The Recall metric indicates the proportion of positive samples that are correctly identified, i.e., it can be used to discriminate the model’s ability in DR grading. We find that our method outperforms other models on Recall by 3 to 8 percentage points, which suggests that our model has better results for different levels of DR. In Fig. 7, on the left is the ROC plot of our model on each category of the APTOS dataset, and on the right is the ROC plot of each model. As can be seen from the figure, our model also competes well on the AUC metric.

**Table 3:** Experimental results on APTOS dataset.

Method	Acc	F1	Precision	Recall	Auc
Resnet50^32^	82.81	66.86	70.13	64.68	80.06
Densenet121^26^	83.21	64.63	67.65	63.52	79.57
Res2Net^33^	82.12	62.60	69.42	59.90	77.57
Swin^34^	81.44	60.58	64.43	59.42	77.28
FasterNet^35^	82.67	65.84	**72.33**	62.91	79.17
SMT^36^	82.53	62.65	71.80	59.39	77.28
FasterViT^37^	81.17	61.51	68.01	59.05	77.02
CoCs^38^	81.58	63.71	68.07	61.53	78.33
VIG^27^	82.81	66.48	69.42	64.52	80.01
**Ours**	**84.31**	**69.69**	72.27	**67.84**	**81.89**

Significant values are in bold.

**Figure 8:**
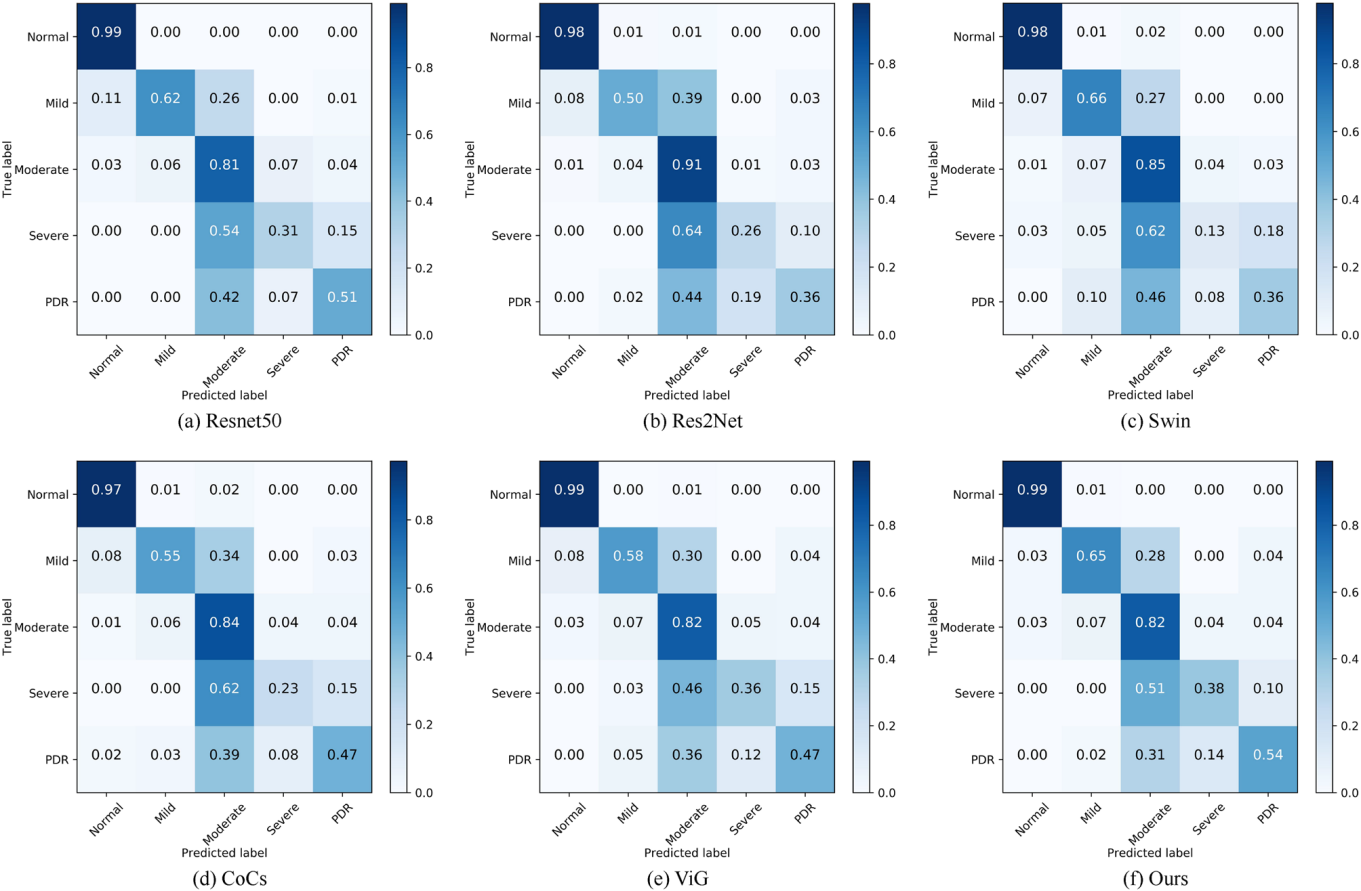
Confusion matrix of six models on APTOS dataset.

To further analyze the specific classification effect of our model on each category on the APTOS dataset, we give the Precision, Recall, and F1 evaluation metrics of the model on the five categories of Normal, Mild, Moderate, Severe, and PDR in Table 4. From Table 4, we can find that the indicators of our model in the Normal category are very good, which indicates that the diagnosis of whether the patient has DR is very accurate, which is conducive to the appropriate treatment at an early stage. And except for the Normal and Moderate categories, the results of other categories are not very good, especially the Severe category has the relatively worst recognition. To analyze the reason for this phenomenon, we draw the confusion matrix of Resnet^32^, Res2Net^33^, Swin^34^, CoCS^38^, ViG^27^, and Ours. As shown in Fig. 8, we can find that each model has the best recognition for Normal and Moderate categories. The recognition effect for the Severe category is the worst among all the categories. Among them, Swin’s probability of correctly identifying Severe is only 13%, relatively speaking, our method has the best recognition effect among all models. The reason for the above occurs because the training datasets for Mild, Severe, and PDR are very small. As can be seen from Table 2, the APTOS dataset has only 154 training images for the Severe category, which is the least among all the categories, and thus its recognition effect is also the worst. Further analyzing the confusion matrix in the figure below, we can see that the main reason for the recognition errors of the categories other than the PDR category is to identify them as neighboring categories. For example, the CoCs^38^ discriminated 34% of the Mild category as Severe and 62% of the Severe categories as Moderate. The reason for this situation may be that the difference between the DR images of the neighboring categories is very small, which is what leads to the misidentification of all the models.

**Table 4:** Recognition effectiveness of our method in each category of the APTOS dataset.

Class	Precision	Recall	F1
Normal	0.98	0.99	0.98
Mild	0.75	0.64	0.69
Moderate	0.73	0.82	0.77
Severe	0.47	0.38	0.42
PDR	0.68	0.54	0.60

####  Results on DDR dataset

**Figure 9:**
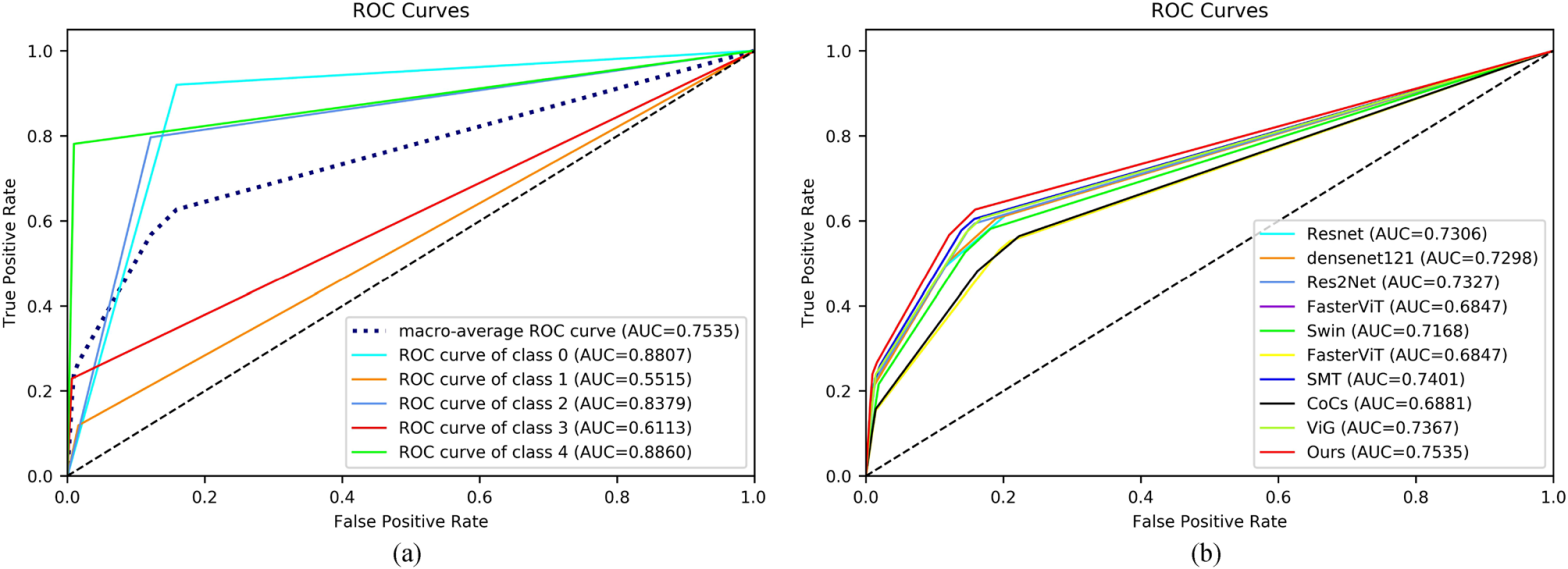
ROC curve on the dataset DDR. (**a**) is the ROC curve for each category of our model, and (**b**) is the ROC curve for each model.

**Figure 10:**
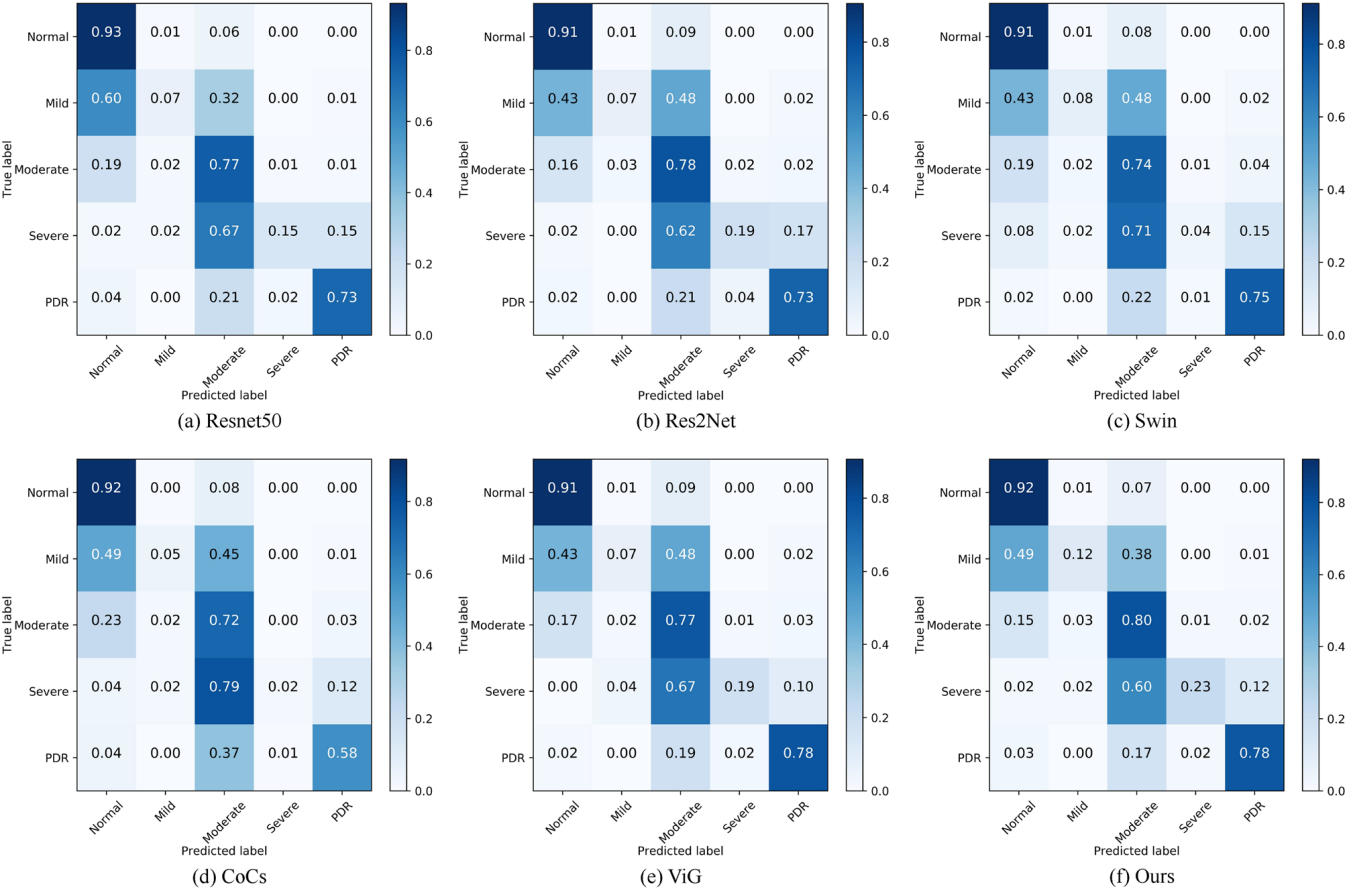
Confusion matrix of six models on APTOS dataset.

We perform experiments on a larger dataset DDR to verify the generalization performance of our model. Table 5 shows the comparison experiments on the dataset DDR. From the table, we can see that our method also achieves significant results compared to the baseline model and other state-of-the-art models. Our method is 81.25% on ACC, 59.18% on F1, 63.91% on Precision, 56.93% on Recall, and 75.34% on AUC. In Fig. 9 we visualize the roc curves for each category of our model and the roc curves for all models on the DDR dataset. Compared to the dataset APTOS, we can see that all models are much lower on all metrics. This is mainly due to the unbalanced distribution of the DDR dataset categories. From Table 2 we can see that the Normal category of the DDR dataset accounts for half the number of the training set, while the Severe category only accounts for 1.87%. Table 6 lists the Precision, Recall, and F1 of our method on each category, while Fig. 10 gives the confusion matrix of some models. Taken together, it can be seen that the recognition ability of each model in Mild and Severe is very bad. This is because the problem of category imbalance is more severe in the DDR dataset compared to the APTOS dataset.

**Table 5:** Experimental results on DDR dataset.

Method	Acc	F1	Precision	Recall	Auc
Resnet50^32^	80.05	55.26	62.05	52.99	73.05
Densenet121^26^	80.21	55.39	61.79	52.69	72.98
Res2Net^33^	79.13	54.78	58.33	53.39	73.27
Swin^34^	78.18	51.11	54.39	50.61	71.68
FasterNet^35^	79.73	54.73	63.18	54.38	73.69
SMT^36^	75.58	45.14	45.40	45.32	68.47
FasterViT^37^	79.17	57.17	61.97	54.73	74.00
CoCs^38^	76.26	47.05	60.05	45.78	68.80
VIG^27^	79.25	55.61	60.11	54.23	73.66
**Ours**	**81.25**	**59.18**	**63.91**	**56.93**	**75.34**

Significant values are in bold.

**Table 6:** Recognition effectiveness of our method in each category of the DDR dataset.

Class	Precision	Recall	F1
Normal	0.85	0.92	0.88
Mild	0.28	0.12	0.16
Moderate	0.78	0.79	0.79
Severe	0.40	0.22	0.29
PDR	0.86	0.78	0.82

**Figure 11:**
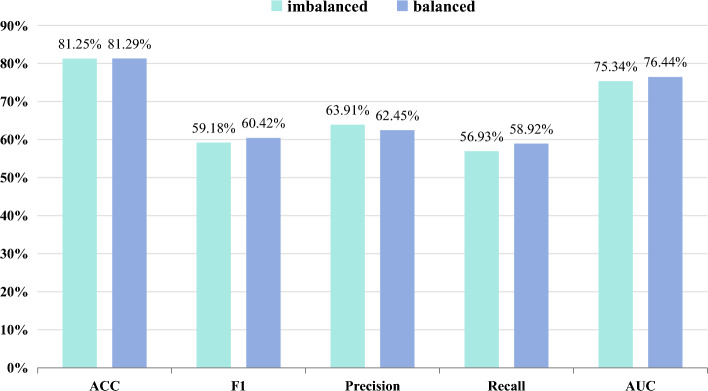
Overall performance change after balancing the DDR dataset.

**Figure 12:**
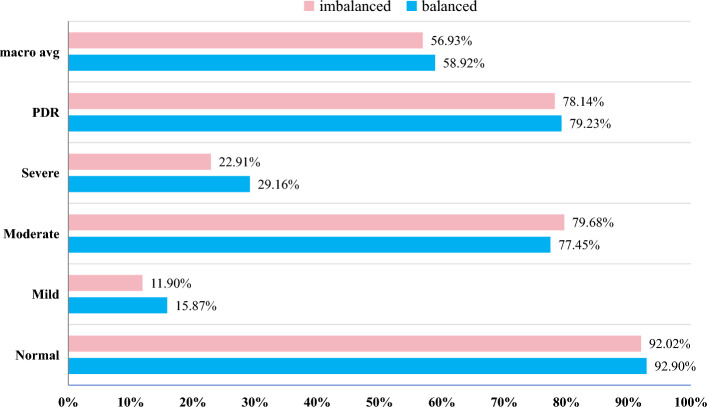
Changes in recall metrics for each category after balancing the DDR dataset.

The effect of the above experiments can be found in the fact that for extremely unbalanced categories, all the models are very bad at recognizing them. The smaller the number of categories, the worse the ability of their models to recognize them. We manually expand the images of some categories to observe whether the recognition ability of the models for each category changes. As can be seen from Table 2, the categories with unbalanced data are Mild, Severe, and PDR. To reduce the inclusion of extra information, we only expand the images by rotating the original training dataset. Table 7 shows the DDR dataset before and after balancing. Fig. 11 shows the comparison of our model in ACC, F1, Precision, Recall, and AUC before and after balancing the dataset. We can find that the change in ACC metrics after balancing the dataset is not significant but its F1 and AUC metrics are improved, which indicates that the model’s recognition ability on categories with fewer images has improved. In Fig. 12 we plot the model’s Recall metric change on each category. As can be seen from Fig. 12, the Recall metrics of Mild, Severe, and PDR with added images are all improved, with the improvement in the Mild and Severe categories being more obvious. The above experiment proves that using certain data balancing techniques can increase the model’s ability to recognize each category.

**Table 7:** Number of DDR training sets before and after balancing.

train	Normal	Mild	Moderate	Severe	PDR	Total
Imbalanced	5012	504	3581	188	730	10015
Balanced	5012	1008	3581	940	1460	12001

### Heatmap analysis

**Figure 13:**
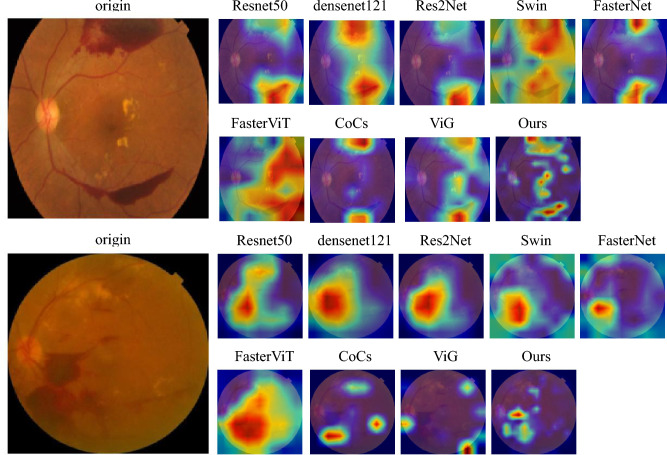
Heat maps for different models. The top image is selected from the APTOS dataset and the bottom image is from the DDR dataset.

In this paper, we use the Grad-CAM^39^ method to generate the corresponding heat maps. The heat map can help us to analyze the area of interest of the network for a certain category, and then we can analyze whether the network learns the right features or information through the area of interest of the network. We selected a picture from the APTOS test set and the DDR test set respectively, and then made heat maps for all models. From Fig. 13, we can find that Resnet50^32^, Densenet121^26^, and Res2Net^33^ can focus on more obvious lesion regions, but they ignore some other small lesion information, and their focus area is large, and they are insensitive to the boundary part of the lesion. Swin^34^ and FasterViT^37^ focus on a part that occupies a large portion of the whole image, and they are also imprecise in recognizing lesion regions. CoCs^38^ and ViG^27^ are relatively accurate in focusing on lesion features, but they also ignore some other categories of feature information. As can be seen from the figure, our method not only focuses on important feature information but also has the best recognition of the boundary of the lesion, which is not easily interfered with by other background information. This is mainly because our model adopts the multi-scale dynamic fusion (MSDF) module, which can identify the information of lesions of different shapes and sizes. By taking advantage of graph convolution, similar features can be feature-converged, which can better focus on its important features.

### Ablation study

#### Model input resolution size

We can find that many of the lesion regions in the DR dataset are relatively small and do not make up a large percentage of the overall image. Therefore we increase the resolution of the model’s input images to see the changes in the model’s DR grading ability. We increased the input images from 224 × 224 to 288 × 288 and 352 × 352, respectively, and kept other settings constant. Table 8 shows the individual metrics of our model for different initial input sizes. As can be seen from Table 8, the overall performance of the model can be improved by increasing the resolution of the input image. On both APTOS and DDR datasets, all the metrics of the image with input size 288 × 288 are better than 224 × 224, and the overall recognition of 352 × 352 is also better than 288 × 288 in all cases.

**Table 8:** Ablation experiment with initial input image size.

Datasets	Size	ACC	F1	Precision	Recall	AUC
APTOS	224	84.31	69.69	72.27	67.84	81.89
	288	84.72	70.91	72.77	69.54	82.78
	352	**85.94**	**71.29**	**74.10**	**69.55**	**82.96**
DDR	224	81.25	59.18	63.91	56.93	75.34
	288	83.04	61.63	67.32	59.16	76.70
	352	**83.88**	**64.27**	**68.72**	**61.68**	**78.20**

Significant values are in bold.

#### Design of MFE

**Figure 14:**
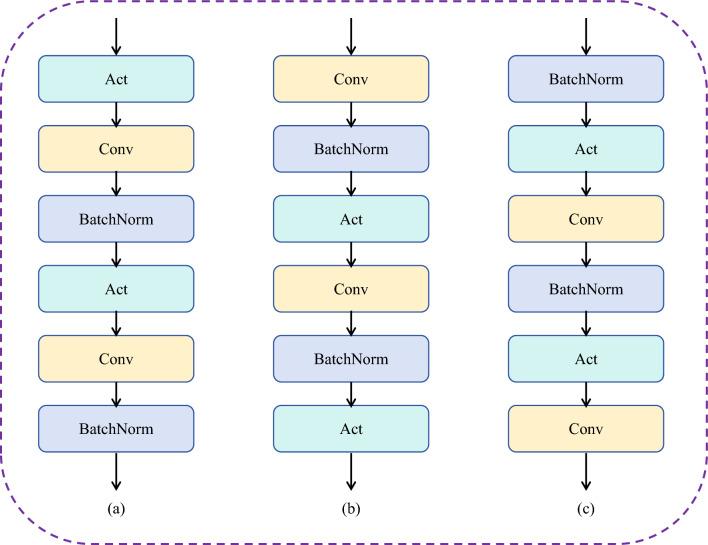
Three ways of designing MFF modules.

In the MFE module, each branch is designed with the order of computation in Fig. 14a, i.e. activation function, then convolution and BatchNorm. And in Fig. 14b is convolution followed by the BatchNorm and activation function. The order of computation in Fig. 14c is BatchNorm, activation function, and convolution. Table 9 shows the experimental results of different branching designs. On the APTOS dataset, the overall effect of adopting the computational order of Fig. 14a is the best, with the highest metrics in ACC, F1, Recall, and AUC. We analyze the reasons why the effect of adopting Fig. 14a is better than that of Fig. 14b and c. First, the activation function used in this paper is GELU, whose outputs are all non-negative, which may constrain the ability of the MFE module to extract multi-scale feature information. Secondly, the role of BatchNorm is to give the data a fixed distribution, so that the distribution of individual features in the same batch is similar, which is conducive to improving the overall performance of the model. Whereas the output of Fig. 14b ends up going through an activation function, Fig. 14c ends up with a convolution, which will diminish the effectiveness of our MFE module. As for the DDR dataset, the gap in accuracy is relatively not very obvious, which may be because the data imbalance problem is more serious in DDR, so it has a limited impact on the overall performance. As for other metrics, the effect of the model using Fig. 14a relatively achieves a better balance and better overall performance.

**Table 9:** Ablation experiments with the MFE design method.

Datasets	Methods	ACC	F1	Precision	Recall	AUC
APTOS	a	**84.31**	**69.69**	72.27	**67.84**	**81.89**
	b	83.76	68.61	**75.37**	65.40	80.48
	c	83.90	68.16	73.71	65.16	80.40
DDR	a	81.25	**59.18**	63.91	**56.93**	**75.34**
	b	**81.49**	58.07	65.16	55.44	74.57
	c	**81.49**	56.77	**67.92**	54.15	73.87

Significant values are in bold.

#### Effectiveness of modules

We implemented a series of ablation experiments on APTOS and DDR datasets to validate the effectiveness of our proposed method. Firstly we experimented with the baseline model ViG^27^ on both datasets. Second, we add the MFE module to the baseline model to verify its effectiveness. Since the MFE module has multiple output features, we experiment with fusing each feature using add and concat respectively. Finally, we add both MFE and MFF modules to the model for experimentation. Table 10 shows the results of the correlation ablation experiments. We can see that compared to the baseline model, the model with only the MFE module added has a considerable effect improvement in each metric. This suggests that using the MFE module to extract multi-scale lesion information is beneficial for the final DR grading. However, for different levels of DR, not every lesion information has equal importance. For example, for PDR images, which may contain lesion features such as neovascularisation, hemorrhagic spots, hard exudates, etc., the most important basis for classifying them in the PDR category is information such as neovascularization. As a result, we designed the MFF module to dynamically fuse multiple output features of the MFE module according to the importance of different features to model final grading. In Table 10 we can find that the model with added MFE and MFF modules has the best overall performance on both datasets.

**Table 10:** Results of ablation experiments with innovative modules.

Datasets	Methods	ACC	F1	Precision	Recall	AUC
APTOS	Baseline(VIG)	82.81	66.48	69.42	64.52	80.01
	Baseline+MFE+ADD	83.62	68.68	71.89	66.49	81.06
	Baseline+MFE+Concat	83.76	68.11	**73.48**	65.35	80.49
	Baseline+MFE+MFF	**84.31**	**69.69**	72.27	**67.84**	**81.89**
DDR	Baseline(VIG)	79.25	55.61	60.11	54.23	73.66
	Baseline+MFE+ADD	80.73	56.61	62.85	56.19	74.82
	Baseline+MFE+Concat	80.81	56.05	**66.55**	54.94	74.16
	Baseline+MFE+MFF	**81.25**	**59.18**	63.91	**56.93**	**75.34**

Significant values are in bold.

## Conclusions

In this paper, we combine the advantages of convolution and graph convolution to propose a novel network for DR grading. We first analyze the characteristics of DR images and the difficulties of DR grading and then propose the corresponding solutions according to these problems. Specifically, the following is discussed. Firstly, the shape of lesion areas in DR images is irregular and their sizes are flexible, so a single feature extractor cannot effectively extract information about lesions of different sizes and shapes. To address this problem, we designed a multi-scale feature extraction (MFE) module, which can extract lesion feature information of different sizes using convolution kernels of different sizes. Secondly, for images above the moderate DR level, there are generally multiple categories of lesion information in the image, but the contribution of this lesion information to the final DR grading is not the same. For this reason, we propose a feature fusion module that dynamically learns the weights for feature fusion based on the connections between the features themselves and other features. Finally, we find that the DR lesion information is found to be distributed throughout the image, suggesting that the model needs to have the ability to model long-range dependencies. We address the whole problem by introducing graph convolution into DR grading. In summary, we propose a novel model MDGNet for DR grading. Extensive experiments show that our proposed method achieves superior performance on both APTOS and DDR datasets. Although our proposed method achieves the best recognition results on both datasets compared to other models, it is found through experiments that the recognition accuracy of our method for certain categories still needs to be improved. Therefore, we will further improve the model in the future to enhance its ability to recognize unbalanced categories.

## Data Availability

The APTOS and DDR datasets are openly available at: https://www.kaggle.com/competitions/aptos2019-blindness-detection (accessed on 12 October 2023) and https://github.com/nkicsl/DDR-dataset (accessed on 12 October 2023).
